# NLRP3 inflammasome activation plays a carcinogenic role through effector cytokine IL-18 in lymphoma

**DOI:** 10.18632/oncotarget.21010

**Published:** 2017-09-18

**Authors:** Xia Zhao, Chen Zhang, Mingqiang Hua, Ruiqing Wang, Chaoqin Zhong, Jie Yu, Fengjiao Han, Na He, Yanan Zhao, Guoqiang Liu, Ni Zheng, Chunyan Ji, Daoxin Ma

**Affiliations:** ^1^ Department of Hematology, Qilu Hospital, Shandong University, Jinan, China; ^2^ Department of Hematology, Shengli Oilfield Central Hospital, Dongying, China; ^3^ Department of Clinical Laboratory, Shengli Oilfield Central Hospital, Dongying, China

**Keywords:** NLRP3, inflammasome, IL-18, lymphoma, mechanism

## Abstract

Inflammasomes play important roles in the pathogenesis of tumors, but the roles of NLRP3 inflammasome in the lymphoma remain unclear. Activated NLRP3 inflammasome induces the maturation of its effector cytokine IL-18 which functions in the development of cancer. Here, we investigated the polymorphism and expression of NLRP3 inflammasome related genes and explored their function in lymphoma. We found that IL-18 (rs1946518) and NFκB94 ins/del (rs28362491) contributed to lymphoma susceptibility and allele G in IL-18 was significantly associated with the risk of lymphoma. The mRNA and plasma expression levels of IL-18 were significantly elevated in primary lymphoma patients and decreased after remission. NLRP3 inflammasome could be activated by ATP plus LPS in lymphoma cells accompanied with the increasing expression of NLRP3-related genes. NLRP3 inflammasome activation reduced the dexamethasone-induced proliferation-inhibiting effect by promoting cells into S phase. NLRP3 inflammasome activation promoted lymphoma cells proliferation and inhibited apoptosis through up-regulation of c-myc and bcl-2, and down-regulation of TP53 and bax, and then reduced the anti-tumor effect of dexamethasone. Similar with the activation of NLRP3, the effector cytokine IL-18 also had the proliferation-promoting, apoptosis-inhibiting and resistance-reducing effects on lymphoma cells via shifting the balance of c-myc/TP53 and bcl-2/bax. Moreover, neutralizing IL-18 has the opposite effects. In conclusion, NLRP3 inflammasome contributes to the susceptibility and plays a carcinogenic role through its effector cytokine IL-18 in lymphoma.

## INTRODUCTION

Lymphoma is a malignant tumor originating from lymphatic hematopoietic system, including Non-Hodgkin’s lymphoma (NHL) and Hodgkin’s lymphoma (HL). NHL is a diverse group of lymphoid proliferative disorders and makes up 90% of all solid tumors of the immune system [1]. Though the therapeutic strategy has made great progress, there are still many patients that fail to achieve remission and relapse at last. The pathogenesis of development of lymphoma is complex. However, the abnormalities in immunological mechanisms have been considered critical in the progress of lymphoma.

Inflammasomes, newly-recognized immune complex, are involved in innate immune recognition of pathogen-associated molecular patterns as well as intracellular and extracellular damage-associated molecular patterns [2]. Recently, more than 20 inflammasomes have been identified, and NLRP3 is the most-studied inflammasome. NLRP3 inflammasome is composed of the NLRP3 molecule, the adaptor ASC (apoptosis-associated speck-like protein) and caspase-1 which can be recruited, assembled and activated by exogenous pathogen invasion or *in vivo* cell damage and death [3]. NLRP3 inflammasome is activated by a two-step deubiquitination mechanism initiated by Toll-like receptor signaling and mitochondrial reactive oxygen species. The first step triggered by pattern-recognition receptors is a priming step typically which leads to the expression of pro-IL-1β and NLRP3; and the second step induced by various ligands is the assembly and activation of the inflammasome molecular complex [4]. NLRP3 inflammasome induces maturation of IL-1β and IL-18 via cleavage of their inactive precursor forms (pro-IL-1β and pro-IL-18) by caspase-1. Active IL-1β and IL-18 can regulate the differentiation of tumor and T helper (Th) cells through downstream signal molecules including NFκB and IL-6 [5].

NLRP3 inflammasome plays important roles in the etiology of inflammation and cancers. As the core of the inflammatory response, NLRP3 inflammasome may have dual effects on tumorigenesis. Recent studies have identified that inflammasome activation has potent antitumorigenic effects by promoting the maturation and release of the IL-1β family, and inducing apoptosis of the tumor precursor [6]. It was reported that NLRP3 activation could down-regulate IL-18-dependent IL-22 binding protein expression, leading to tissue damage and a protective effect when tissue damage to a certain extent, thereby promoting tissue repair and inhibiting tumorigenesis [7]. However, a broad range of experimental and clinical evidence supports the role of inflammation in the promotion of tumor growth in recent years. Inflammasomes release a large number of IL-1 and IL-18 and other inflammatory cytokines through the NLRP3/caspase axis to promote tumorigenesis. The inhibition of inflammasomes or neutralization of their products, mainly IL-1β and IL-18, has profound inhibiting effects on carcinogenesis and tumor progression [8]. Moreover, the activation of inflammasome was related to migration and metastasis of tumor cells such as gastric cancer, skin cancer, prostate cancer, liver cancer, breast cancer and other tumor cells [9–12].

Moreover, functional single nucleotide polymorphisms (SNPs) affect the inflammatory microenvironment nesting malignant tumors [13], and SNPs may also provide a direct effect on the malignant cells [14]. Genetic SNPs of the genes associated with NLRP3 inflammasome were found to be related to several cancers. IL-18 gene polymorphisms were associated with prostate cancer and its recurrence [15]. IL-1β polymorphisms were also linked to several kinds of malignant tumors, such as gastric cancer, hepatocellular carcinoma and lung cancer [16–18].

Thus, inflammasomes are promising therapeutic targets in cancer-related clinical conditions. However, the role of NLRP3 inflammasome in the development and treatment of lymphoma has not been reported until now. In this study, we investigated the polymorphism and expression of NLRP3 inflammasome related genes in lymphoma patients and evaluated their clinical relevance. Moreover, we further explored their function and clarified their mechanisms in lymphoma.

## RESULTS

### IL-18(rs1946518) and NFκB 94 ins/del (rs28362491)contributed to lymphoma susceptibility

SNPs genotypic frequencies of NLRP3 related genes were in the Hardy-Weinberg equilibrium in both patients and controls. Both IL-18 (rs1946518)(χ^2^ = 136.0579, *P* < 0.0001) and NFκB 94 ins/del (rs28362491) (χ^2^ = 6.1462, *P* = 0.0463) resulted to be significantly associated with lymphoma. Analysis of the alleles of IL-18 (rs1946518) showed a significantly increased risk in patients carrying the allele “G” in all subjects in dominant model (OR = 3.858, 95 % CI = 3.066–4.854, *P* < 0.0001). Also the “ins” allele carrier of NFκB 94 ins/del was associated with increased risk for lymphoma (OR = 1.241, 95 % CI = 1.013–1.520, *P* = 0.0371). There was no significant difference in the distribution of IL-1β (rs16944) (χ^2^ = 1.6345, *P* = 0.4416) and CARD8 (rs2043211) (χ^2^ = 1.4322, *P* = 0.4887) polymorphisms between lymphoma patients and controls (Table 1).

**Table 1:** Genotype distribution of the different polymorphisms in lymphoma patients and normal control population investigated in the study and their association with lymphoma susceptibility

SNPs	Case *n* (%)	Control *n* (%)	χ^2^	*P*	OR (95% CI)
**IL-18 (rs1946518)**			**136.0579**	**< 0.0001**	
** GG**	**264 (67.69)**	**104 (27.01)**			
** GT**	**108 (27.69)**	**203 (52.73)**			
** TT**	**18 (4.62)**	**78 (20.26)**			
**Allelic frequency**					
** G**	**636 (81.54)**	**411 (53.37)**			**3.858 (3.066–4.854)**
** T**	**144 (18.46)**	**359 (46.63)**	**140.1924**	**< 0.0001**	
IL-1β(rs16944)			1.6345	0.4416	
GG	117 (30.00)	100 (25.97)			
GA	178 (45.64)	189 (49.09)			
AA	95 (24.36)	96 (24.94)			
Allelic frequency					
G	412 (52.82)	389 (50.52)			1.097 (0.898–1.338)
A	368 (47.18)	381 (49.48)	0.8216	0.3647	
CARD8(rs2043211)			1.4322	0.4887	
AA	97 (27.40)	118 (30.65)			
AT	173 (48.87)	172 (44.68)			
TT	84 (23.73)	95 (24.67)			
Allelic frequency					
A	367 (51.84)	408 (52.99)			0.955 (0.778–1.171)
T	341 (48.16)	362 (47.01)	0.1959	0.6581	
**NFκB-94 (ins/del ATTG)**			**6.1462**	**0.0463**	
** ins/ins**	**151 (39.43)**	**119 (30.91)**			
** ins/del**	**163 (42.56)**	**189 (49.09)**			
** del/del**	**69 (18.01)**	**77 (20.00)**			
**Allelic frequency**					
** ins**	**465 (60.70)**	**427 (55.45)**			**1.241 (1.013–1.520)**
** del**	**301 (39.30)**	**343 (44.55)**	**4.3476**	**0.0371**	

Statistical significant *P* values and corresponding lines are displayed in bold.

**Figure 1:**
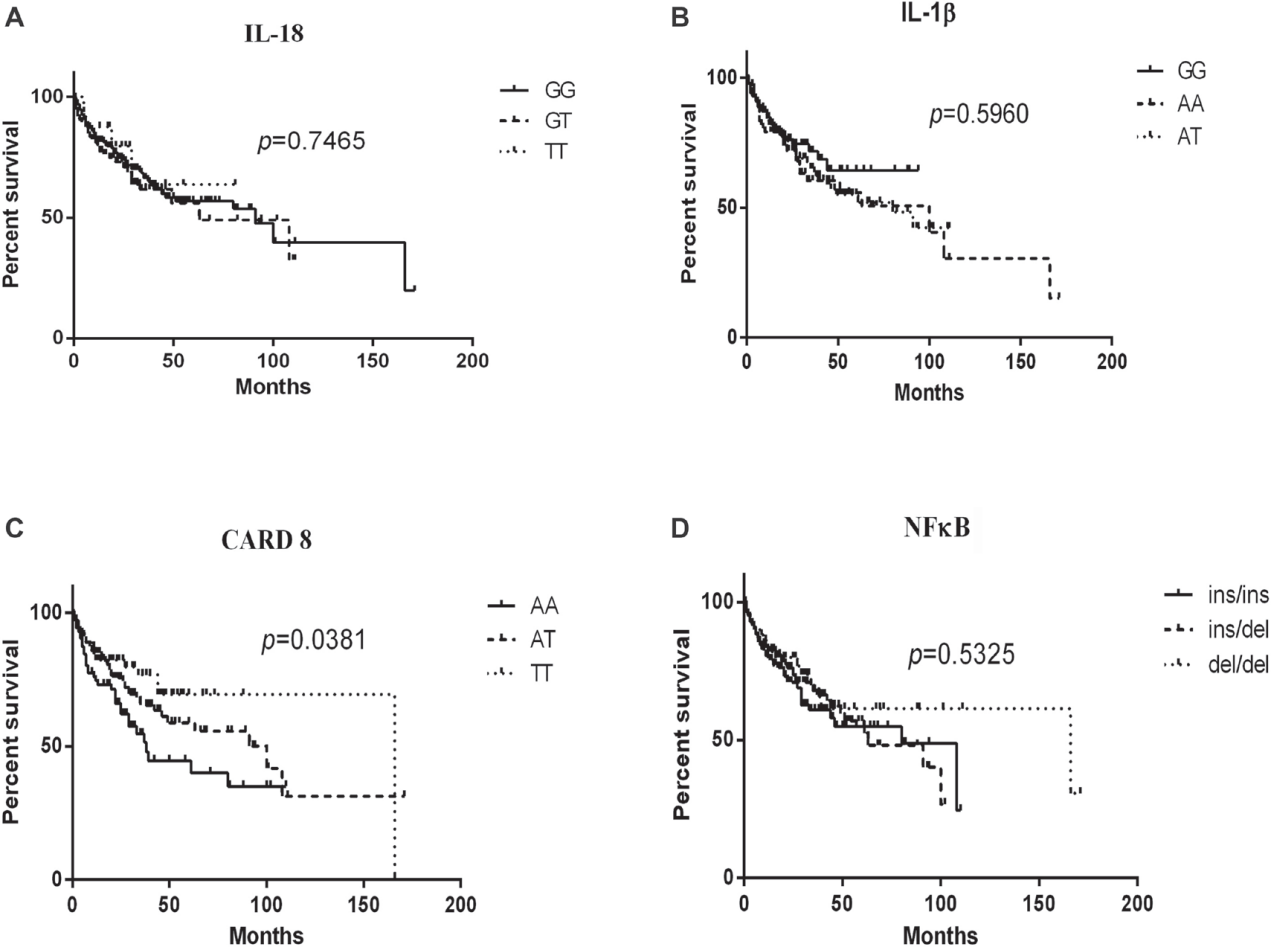
Kaplan-Meier curves of IL-18 (rs1946518), IL-1β (rs16944), CARD8 (rs2043211) and NFκB-94 ins/del ATTG genotypes in lymphoma patients Log-rank *P* values compared with the different genotypes are displayed, and CARD8 (rs2043211) AA genotype contributed to poorer lymphoma-specific survival.

For each SNP, patients were stratified according to their specific genotypes and the survival data was described in Figure 1. The AA genotype of CARD8 (rs2043211) led to a statistically poorer lymphoma-specific survival (*P* = 0.0381). No significant difference was found for the overall survival of patients with IL-18 (rs1946518) (*P* = 0.7465), NFκB -94 ins/del ATTG (*P* = 0.5325) or IL-1β (rs16944) (*P* = 0.5960).

### IL-18 (rs1946518) GT genotype contributed to the higher level of IL-18 in lymphoma patients

**Figure 2:**
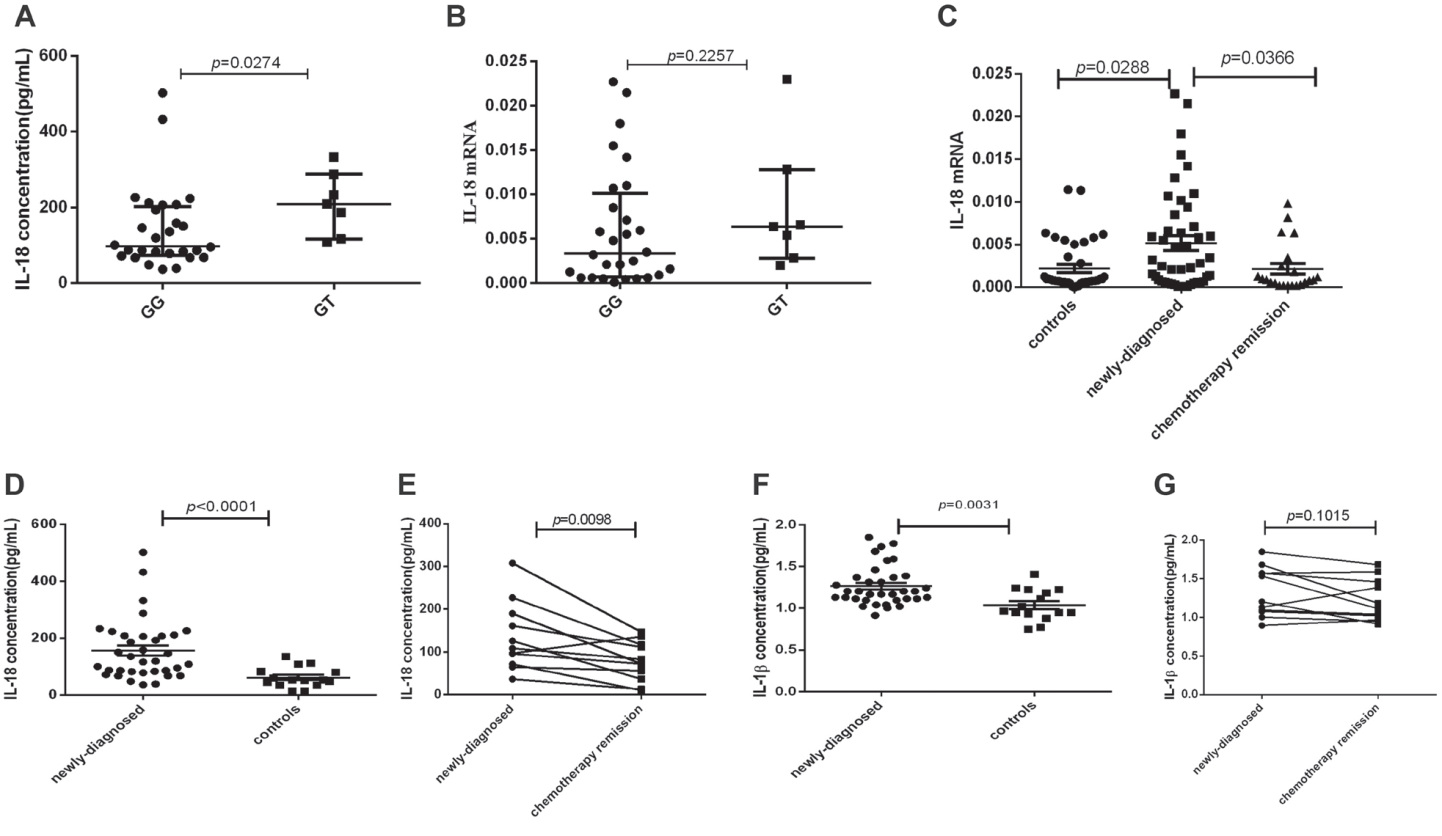
The mRNA expression and plasma levels of IL-18 and IL-1β in lymphoma patients and controls were detected by RT-PCR and ELISA (**A**) IL-18 plasma level in GT and GG genotype of IL-18 (rs1946518) in sample-matched 35 lymphoma patients. (**B**) IL-18 mRNA expression in GT and GG genotype of IL-18 (rs1946518) in sample-matched 35 lymphoma patients. (**C**) IL-18 mRNA expression in sample-available newly-diagnosed, relieved lymphoma patients and controls. (**D**) Plasma IL-18 level in newly-diagnosed lymphoma patients and controls. (**E**) Plasma IL-18 level in newly-diagnosed lymphoma patients before and after chemotherapy remission. (**F**) Plasma IL-1β level in lymphoma patients and controls. (**G**) Plasma IL-1β level in lymphoma patients.

Thirty-five sample-matched newly-diagnosed lymphoma patients were included to explore the relationship between IL-18 genotype and its expression. The results showed that IL-18 plasma level of IL-18 (rs1946518) GT genotype patients was statistically higher than that of GG genotype (*P* = 0.0274, Figure 2A). Though IL-18 mRNA expression has the same trend for all the genotypes, no significant difference was found (*P* = 0.2257, Figure 2B).

### IL-18 expression was increased in newly-diagnosed lymphoma patients and down-regulated after chemotherapy remission

To study the expression profile of IL-18 in lymphoma patients, we selected 68 sample-available lymphoma patients including 46 newly-diagnosed patients and 22 relieved patients and 40 controls for mRNA detection by RT-PCR and 35 lymphoma patients and 15 controls for plasma detection by ELISA. Our study found that IL-18mRNA expression was significantly higher in newly-diagnosed lymphoma patients than in controls (*p* = 0.0288), and decreased in relieved lymphoma patients after chemotherapy (*p* = 0.0366, Figure 2C). Accordingly, plasma IL-18 level was also significantly elevated in newly-diagnosed lymphoma patients than in controls (*p* < 0.0001, Figure 2D), and decreased after chemotherapy remission in the paired 11 patients (*p* = 0.0098, Figure 2E). As for another NLRP3 effector molecule, though plasma IL-1β level in newly-diagnosed lymphoma patients was significantly higher than in controls (*p* = 0.0031, Figure 2F), no significant decrease was found after chemotherapy remission (*p* = 0.1015, Figure 2G).

### NLRP3 inflammasome in lymphoma cells was primed and activated by ATP plus LPS

**Figure 3:**
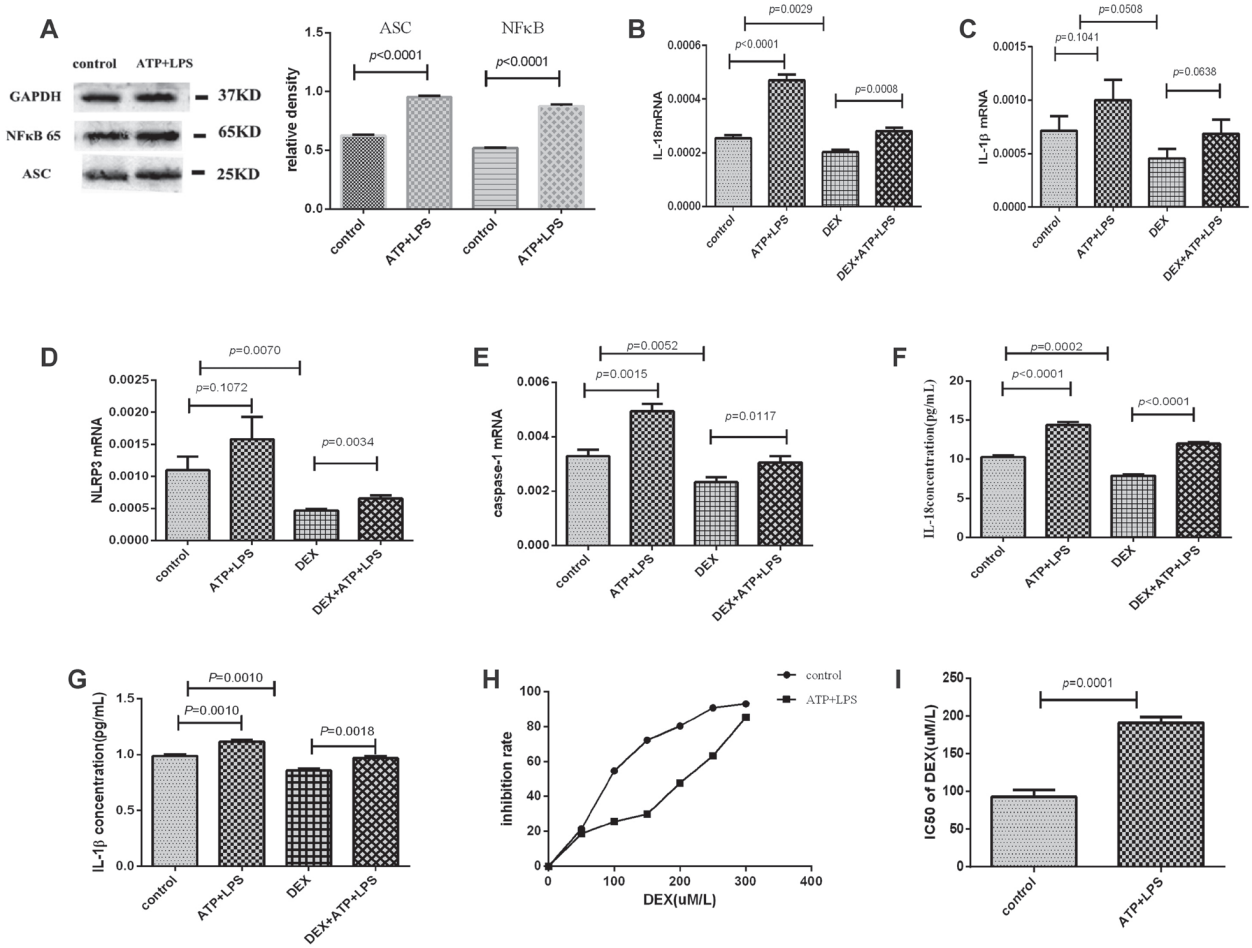
NLRP3 inflammasome was activated by ATP plus LPS, and NLRP3 inflammasome activation enhanced the resistance of Pfeiffer cells to dexamethasone The experimental groups were control, ATP+LPS, DEX and DEX+ATP+LPS. (**A**) Western blot showed that the protein expression levels of ASC and NFκB were elevated after adding ATP and LPS in Pfeiffer cells. (**B**–**E**) The mRNA expression results of IL-18, IL-1β, NLRP3 and caspase-1 in every group. (**F**, **G**) The supernatant levels of IL-18 and IL-1β in every group. (**H**) The results of the inhibition rate of Pfeiffer cells. (**I**) The IC50 value of dexamethasone of Pfeiffer cells before and after adding ATP and LPS.

After being treated with ATP plus LPS, the protein expression level of ASC or NFκB was elevated (*P* < 0.0001), and the mRNA expression levels of IL-18 and caspase-1 were significantly increased in Pfeiffer cells (*P* < 0.01). IL-1β or NLRP3 mRNA expression was also marginally increased (*P* = 0.1041 or 0.1072). These results suggested that NLRP3 inflammasome of Pfeiffer cells was primed by ATP plus LPS. Moreover, in the cultured supernatant of Pfeiffer cells, the plasma levels of IL-18 (*P* < 0.0001) and IL-1β (*P* = 0.001) were significantly increased after adding ATP and LPS, which further suggested that NLRP3 inflammasome was activated and effector molecules were secreted. (Figure 3A–3G).

### NLRP3 inflammasome activation enhanced the resistance of Pfeiffer cells to dexamethasone

After being treated with ATP plus LPS, the inhibition rate of dexamethasone for Pfeiffer cells was down-regulated and the IC50 value of dexamethasone was significantly increased, which suggested that NLRP3 inflammasome activation enhanced the resistance of Pfeiffer cells to dexamethasone (Figure 3H, 3I). In terms of mechanism, the mRNA levels of IL-18, IL-1β, NLRP3 and caspase-1 were significantly decreased after adding dexamethasone, but were significantly elevated after activating inflammasome NLRP3 with ATP plus LPS (Figure 3B–3E). Moreover, the levels of IL-18 and IL-1β in the cultured supernatant of Pfeiffer cells were significantly decreased after adding dexamethasone, while significantly up-regulated after the activation of NLRP3 inflammasome (Figure 3F, 3G). These results further suggested NLRP3 inflammasome activation reduced the effect of dexamethasone, thereby enhancing the resistance of Pfeiffer cells to dexamethasone.

### NLRP3 inflammasome activation reduced the proliferation-inhibiting effect of dexamethasone through promoting cells into S phase

**Figure 4:**
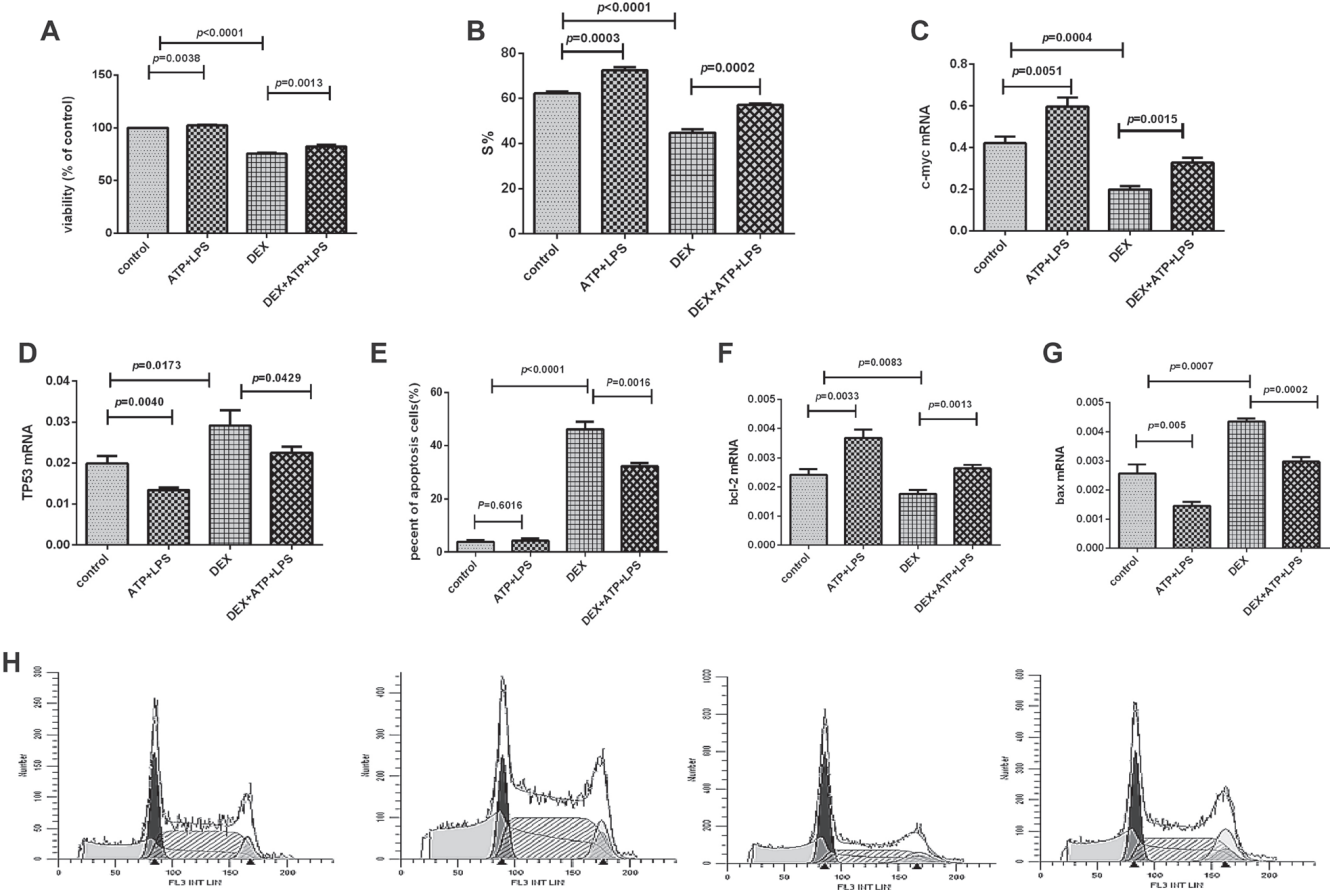
NLRP3 inflammasome activation promoted the Pfeiffer cell proliferation and inhibited the cell apoptosis (**A**) Cell proliferation was detected by CCK8 assay in every group. (**B**) S% of cell cycle was detected by flow cytometry in every group. (**C**–**D**) The mRNA expression results of c-myc and TP53 in every group. (**E**) Cell apoptosis was detected by flow cytometry in every group. (**F**, **G**) The mRNA expression results of bcl-2 and bax in every group. (**H**) Results were plotted as the percentage of cells in each cell cycle phase. The order of the above graphics is as follows: control, ATP+LPS, DEX, and DEX+ATP+LPS.

Dexamethasone suppressed Pfeiffer cells proliferation, but the activation of NLRP3 inflammasome significantly reduced this inhibiting effect and enhanced the cell viability significantly. As to mechanism, our results showed that NLRP3 inflammasome activation promoted the cell cycle from G1 to S phase (Figure 4A, 4B, 4H). Furthermore, in molecular level, the mRNA expression of c-myc was decreased and TP53 was increased significantly in Pfeiffer cells after being treated with dexamethasone, but NLRP3 inflammasome activation reversed the expression levels of these two genes (Figure 4C, 4D).

### NLRP3 inflammasome activation inhibited dexamethasone-induced apoptosis via shifting the balance of bcl-2/bax expression

Dexamethasone induced cell apoptosis, which was significantly reduced when NLRP3 inflammasome was activated, and the apoptosis was significantly decreased (Figure 4E, 5G). After the addition of dexamethasone, the mRNA expression of bcl-2 was significantly decreased, and bax expression was significantly increased. And NLRP3 inflammasome activation largely reversed the effect of dexamethasone (Figure 4F, 4G), that is, bcl-2 mRNA expression was significantly up-regulated and bax expression was significantly down-regulated after NLRP3 was activated.

**Figure 5:**
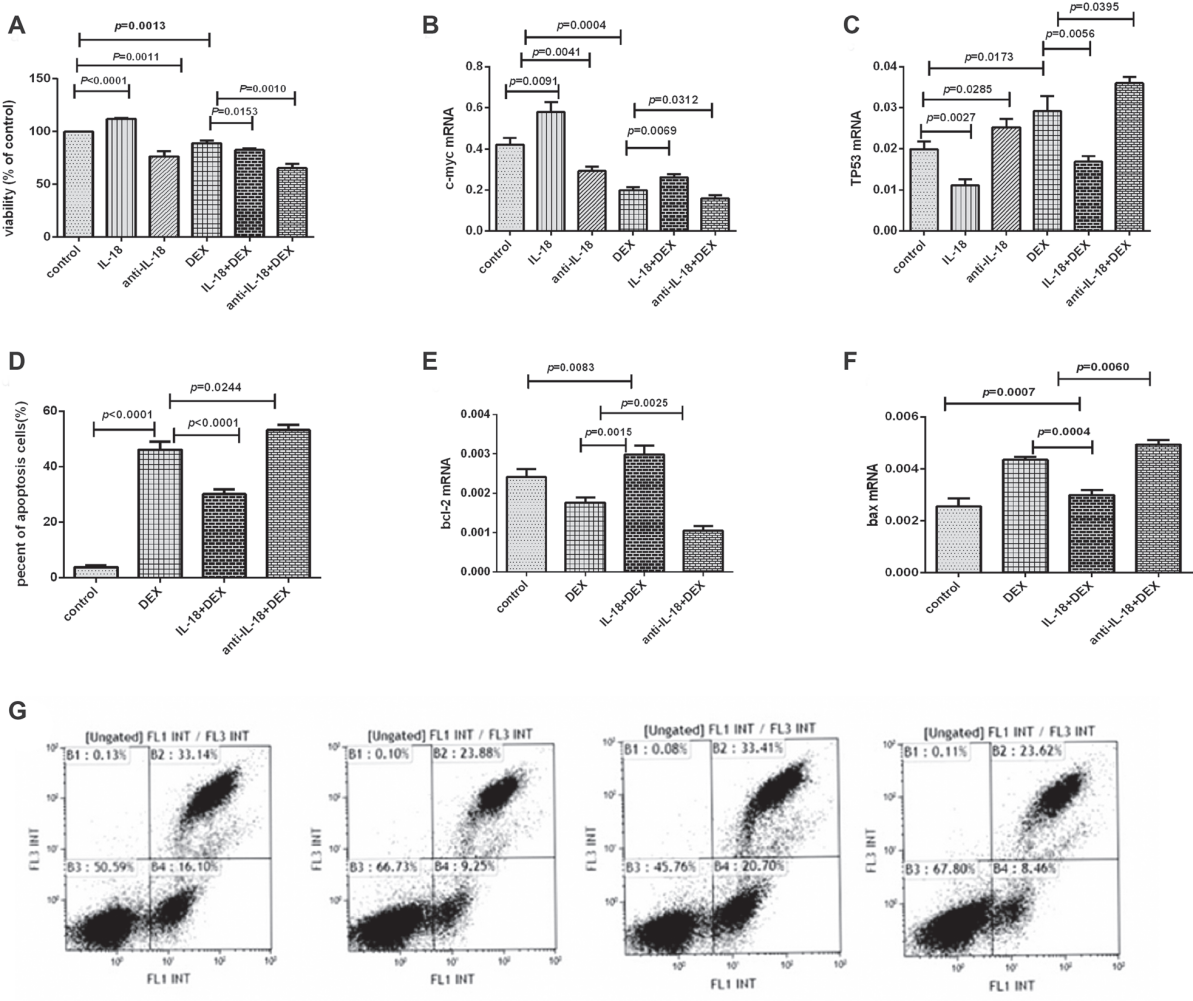
The effects of the IL-18 and anti-IL-18 on lymphoma cells (**A**) Cell proliferation was detected by CCK8 assay in every group. (**B**–**C**) The mRNA expression results of c-myc and TP53 in every group. (**D**) Cell apoptosis was detected by flow cytometry in every group. (**E**, **F**) The mRNA expression results of bcl-2 and bax in every group. (**G**) Results were plotted as the percentage of cell apoptosis. The order of the above graphics was as follows: DEX, DEX+ATP+LPS, IL-18+DEX, and anti-IL-18+DEX.

### IL-18 mediated the biological effects of NLRP3 inflammasome on Pfeiffer cells

We have demonstrated the elevated IL-18 in lymphoma patients was decreased after remission and activated NLRP3 inflammasome could induce higher IL-18. Therefore, after confirming the expression of IL-18R in Pfeiffer cells, we performed functional studies on Pfeiffer cells using IL-18 and its neutralizing antibody. The proliferation of Pfeiffer cells was significantly enhanced after the addition of IL-18, but was significantly inhibited after adding anti-IL-18. Dexamethasone suppressed the cell proliferation, but IL-18 significantly reduced the efficacy of dexamethasone drugs, and the cell proliferation was significantly increased. However, anti-IL-18 significantly enhanced the efficacy of dexamethasone drugs, and the cell proliferation was significantly decreased (Figure 5A). For the molecular mechanism, the mRNA expression of c-myc was significantly increased, while TP53 was decreased after IL-18 was added. However, after the addition of anti-IL-18, c-myc was decreased, and TP53 mRNA expression was significantly increased (Figure 5B, 5C).

Flow cytometry showed that dexamethasone promoted cell apoptosis, but this effect was significantly reduced by IL-18 and enhanced by anti-IL-18 (Figure 5D, 5G). Molecularly, the mRNA expression of bcl-2 was increased, while bax was decreased significantly after IL-18 was added. Moreover, neutralizing IL-18 down-regulated bcl-2 and up-regulated bax expression significantly (Figure 5E, 5F).

## DISCUSSION

Recently, illuminating how the immune system influences tumor development and progression remains one of the most challenging questions in oncology. Studies have found that NLRP3 inflammasome plays an important role in tumor development and chemotherapy resistance. However, the role of NLRP3 inflammasome in the development and therapy of lymphoma has not been reported.

Although NLRP3 inflammasome has been reported to eliminate malignant progenitor cells by programmed cell death [19], more recent studies have shown that NLRP3 inflammasome plays a role in promoting cancer [20]. It is still unclear what cellular signaling events mediate inflammasome assembly and activation. ASC is a necessary component of the fully assembled and active NLRP3 inflammasome [21]. IL-1β, the effector molecule of NLRP3 inflammasome, was found highly expressed in transgenic mice. The transcription factor NFκB was activated by IL-1 receptor to promote tumor development, and the application of IL-1β neutralizing antibodies could significantly inhibit tumorigenesis and development [22]. It is reported that IL-1β could promote tumor growth by inducing the release of downstream molecule IL-6 in patients with multiple myeloma [23]. IL-18, another effector molecule of NLRP3 inflammasome, could inhibit the growth of NK cells to promote tumor growth and metastasis by inducing the expression of PD-1. However, IL-18 became incompetent by RNAi interference or the application of IL-18 binding protein (IL-18BP), which activated NK cells to inhibit tumor growth [8, 24]. Researches have confirmed that chemotherapy drugs could induce the production of NLRP3 inflammasome, activate the caspase-1 molecules and induce secretion of IL-1β, which can inhibit the anti-tumor effect of chemotherapy [25, 26]. Thus, NLRP3 inflammasome and its products can directly promote the growth of tumor cells and drug resistance. Targeting NLRP3 inflammasome is expected as an important means of cancer therapy.

IL-1β is located on chromosome 2q14 and its polymorphism and expression have been found to be associated with various cancers [21, 27]. However, we found no significant difference in the distribution of IL-1β (rs16944) polymorphism between lymphoma patients and controls, and no statistical significance was found for the overall survival of patients with IL-1β (rs16944). Caspase-associated recruitment domains (CARD) are protein-protein interaction modules found extensively in proteins that play important roles in apoptosis, NFκB activation and cytokine regulation. CARD8 represents a new signaling molecule involved in the regulation of caspase-1 and NFκB activation [28]. We found that the AA genotype of CARD8 (rs2043211) led to statistically poorer lymphoma-specific survival.

NFκB is a key controller of the innate immune response in the intestinal epithelium and in infiltrating inflammatory cells. Recent studies have further emphasized the importance of cancer-related inflammation which revealed that tumor growth and progression depending on the activation of NFκB [29, 30]. There are several studies of NFκB-94 ins/del polymorphism in carcinomas and other diseases which found that this polymorphism may influence cancer risk in Asian population. The variant allele of NFκB-94 ins/del ATTG polymorphism was associated with higher risk of sporadic CRC in Malaysian population [31–33]. In our study, we found that NFκB-94 ins/del resulted to be significantly associated with lymphoma, and the “ins” allele carrier of NFκB-94 ins/del was associated with increased risk for lymphoma.

IL-18, belonging to IL-1 super family, is a pleiotropic proinflammatory cytokine with dual effects on tumor development and progression. IL-18 is produced by various immune and non-immune cells, and plays a role in the host's response to oncogenesis and angiogenesis [34, 35]. A pooled analysis found that both IL-18-607(C/A) and-137(G/C) polymorphisms were associated with significant increase in cancer risk. Takada et al. [36] reported that C allele, at position-607, was a risk factor for sarcoidosis in Japanese population. Given the potential roles of IL-18 in the immune system against tumor cells, angiogenesis, metastasis, proliferation and immune escape, the IL-18 polymorphisms may modulate the risk of cancer [37].

In the present study we found the polymorphism of IL-18 (rs1946518) was significantly associated with lymphoma susceptibility and GG genotype in IL-18 is significantly associated with the risk of lymphoma. We also found that the allele “G” of IL-18 (rs1946518) showed a significantly increased risk of lymphoma. We also demonstrated that plasma IL-18 level among lymphoma patients carrying GT genotype was higher than those carrying the GG genotype. We further investigated the expression of IL-18 in lymphoma patients, and found that the mRNA and plasma expression levels of IL-18 in newly-diagnosed lymphoma patients were significantly higher than controls, and decreased significantly after chemotherapy remission. This suggested that IL-18 was highly expressed in lymphoma patients.

Because of the aberrant polymorphism and expression of NLRP3 inflammasome in lymphoma, we further used lymphoma cell line Pfeiffer to explore their function and mechanism. It has been reported that NLRP3 could be activated by LPS and ATP [38]. Our study found that the expression levels of NLRP3-related genes IL-18, IL-1β, NLRP3 and caspase-1, were increased after addition of ATP plus LPS, suggesting NLRP3 could be primed and activated in lymphoma cells. Research has shown that a hallmark of MDS was the activation of NLRP3 inflammasome, which drives clonal expansion and pyroptotic cell death. Independent of genotype, MDS hematopoietic stem and progenitor cells (HSPCs) overexpressed inflammasome proteins and manifested activated NLRP3 complexes that directed activation of caspase-1, generation of IL-1β and IL-18, and pyroptotic cell death [39]. Our study found that the activation of NLRP3 could promote cell proliferation, inhibit cell apoptosis and enhance the resistance of Pfeiffer cells to dexamethasone. For further molecular mechanism, we found that the activation of NLRP3 inflammasome may be due to the up-regulation of oncogenic c-myc and bcl-2, and down-regulation of anti-oncogenic TP53 and bax expression. Moreover, the elevated effector IL-1β could promote formation of the TCF/Lef/β-catenin complex which induces sequential expression of c-myc, leading to up-regulation of proliferation [40].

As IL-18 plays an extremely important role in the pathogenesis and development of lymphoma, we used IL-18 and its neutralizing antibody to further investigate its mechanism. We found that IL-18 could up-regulate c-myc and bcl-2, and down-regulate TP53 and bax expression, thereby promoting lymphoma cell proliferation, inhibiting lymphoma cell apoptosis and reducing the anti-tumor effect of dexamethasone. Moreover, neutralizing IL-18 could down-regulate the expression of c-myc and bcl-2 and up-regulate the expression of TP53 and bax, thereby inhibiting cell proliferation, promoting lymphoma cell apoptosis, and enhancing the anti-tumor effect of dexamethasone.

Therefore, our results suggested that IL-18 (rs1946518) polymorphism may be an unfavorable factor that increases the susceptibility to lymphoma. The activation of NLRP3 may increase the expression of NLRP3-related genes such as IL-18, IL-1β, NLRP3 and caspase-1 in lymphoma cells, thereby promoting proliferation, inhibiting apoptosis, and reducing the anti-tumor effect of dexamethasone. Among them, IL-18 level in the inflammasome pathway plays a more critical role. Up-regulation of IL-18 can activate NLRP3, promote proliferation, inhibit apoptosis, and inhibit the anti-tumor effect of dexamethasone. Neutralizing IL-18 can reverse these effects.

## MATERIALS AND METHODS

### Patients and controls

A total of 390 lymphoma patients (230 males and 160 females; median age, 54 years) were diagnosed according to WHO classification criteria were enrolled from May 2007 to December 2015. All lymphoma patients were treated with a standard induction chemotherapy based on new NCCN guideline. The B-NHL patients that were mentioned in our paper were mainly treated with CHOP (cyclophosphamide, doxorubicin, vincristine, prednisone) regimen with or without rituximab, the T-NHL patients were mainly treated with CHOP (cyclophosphamide, doxorubicin, vincristine, prednisone), and the HL patients were mainly treated with ABVD (doxorubicin, bleomycin, vinblastine, dacarbazine). In order to avoid the drugs influence, we collected blood samples from newly diagnosed patients before chemotherapeutic treatment. And the collection of blood samples from treated patients took place after at least two weeks when they received chemotherapy. The population included 268 patients with B-NHL, 100 patients with T-NHL, and 22 patients with HL. Samples from 385 gender-and age-matched healthy individuals (233 males and 152 females, median age, 48 years) were collected as controls in this study. Clinical characteristics are summarized in Table 2. Ethical approval for the study was obtained from the Medical Ethical Committee of Qilu Hospital, Shandong University. Written informed consent was obtained from all participants.

**Table 2:** Samples characteristics

	Number	
Parameter	lymphoma (*n =* 390)	Control (*n =* 385)
Male: female (ratio)	1.44	1.47
Median age (years)	54(13–85)	48 (21–85)
lymphoma subtypes (WHO)		
B-NHL	268(68.72%)	
T-NHL	100(25.64%)	
HL	22(5.64%)	
Serum LDH, U/L		
Normal	205(52.56%)	
> Normal	185(47.44%)	
Ann Arbor staging		
I + II	71(18.21%)	
III + IV	319(81.79%)	
Bone marrow involvement		
(–)	334(85.64%)	
(+)	56((14.36%)	
B symptoms		
(–)	309(79.23%)	
(+)	81(20.77%)	
IPI		
0–2	249(63.85%)	
3–5	141(36.15%)	

Ages are presented as median (range) and the other characteristics are presented as *n* (%). B-NHL, B-cell non-Hodgkin’s lymphoma; T-NHL, T-cell non-Hodgkin’s lymphoma; HL, Hodgkin’s lymphoma; LDH, Lactate Dehydrogenase; IPI, International Prognostic Index.

### DNA extraction and genotyping

Formalin-fixed paraffin-embedded (FFPE) bone-marrow aspirate or ethylene diamine tetraacetic acid (EDTA) stabilized peripheral blood was used for DNA extraction from all included individuals. Genomic DNA (gDNA) was extracted using a TIANamp blood DNA kit (Tiangen Biotech, Beijing, China) according to manufacturer’s instructions. The detection reagent of three SNPs, IL-1β (rs16944), IL-18 (rs1946518) and CARD8-C10X (rs2043211), were purchased from Thermo Fisher Scientific company (Cat.#4351379). NFκB-94 ins/del ATTG promoter polymorphism was detected using the forward primer: 5′- CCGTGCTGCCTGCGTT -3′, reverse primer: 5′- GCTGGAGCCGGTAGG GAA -3′ as well as probe 1: 5′-VIC- ACCATT GATTGGGCC -MGB-3′ and probe 2: 5′-FAM- CGACCATTGGGCC -MGB-3′. All PCR reactions were run in one time, and contained 3μl TaqMan Universal PCR Master Mix (2×), 0.15ul pre-designed primers and probes (40×), 1 μl of DNA and 1.85 μl ddH2O. Real-time PCR was performed on an ABI 7500 real-time PCR System(SDS, PE Biosystems) using the following conditions: 50°C for 2 min, 95°C for 10 min, and then 40 cycles of amplification (92°C denaturationfor 15 s, 62°C annealing/extension for 60 s). Genotypes were analyzed using ABI 7500 Sequence Detection System (SDS) 1.3.1.

### RNA extraction, cDNA synthesis and quantitative real-time PCR

We selected 68 sample-available lymphoma patients including 46 newly-diagnosed patients and 22 relieved patients and 40 controls for mRNA detection. Total RNA was extracted from peripheral blood mononuclear cells (PBMCs) of 68 lymphoma patients and 40 controls or cultured Pfeiffer cells using Trizol (Invitrogen, USA) according to the manufacturer’s instruction. RNA was subjected to first-strand cDNA synthesis using Prime script RT reagent kit perfect Real Time (Takara BioInc, Japan). Reverse transcription reaction was done at 37°C for 15 min, followed by 85°C for 5 s. Real-time PCR was conducted using Light Cycler 480II real-time PCR system (Roche, Switzerland) in accordance with the manufacturer’s instruction. The real-time PCR contained, in a final volume of 10 μL, 5 μL of 2×SYBR Green Real-time PCR Master Mix, 1 μLof cDNA, 0.8μLof the forward and reverse primers, and 3.2 μL ddH_2_O. PCR primers of related genes were shown in Table 3. All experiments were conducted in triplicate. The PCR products were analyzed by melt curve analysis and agarose gel electrophoresis to determine product size and to confirm that noby-product was formed. The results were expressed relative to the number of GAPDH transcripts used as an internal control.

**Table 3:** The sequences of PCR primers

gene		Primers (5′–3′)
IL-18	F-GCTTGAATCTAAATTATCAGTC	R-GAAGATTCAAATTGCATCTTAT
IL-1β	F-TTACAGTGGCAATGAGGATGAC	R-GTCGGAGATTCGTAGCTGGAT
NLRP3	F-CGTGAGTCCCATTAAGATGGAGT	R-CCCGACAGTGGATATAGAACAGA
caspase-1	F-TGTCCTGGGAAGAGGTAGAA	R-TGCCTGTTCCTGTGATGTGG
ASC	F-CGCGAGGGTCACAAACGT	R-TGCTCATCCGTCAGGACCTT
bcl-2	F-GAACTGGGGGAGGATTGTGG	R-GCCGGTTCAGGTACTCAGTC
TP53	F-GGACGGAACAGCTTTGAGGT	R-CTCCCCTTTCTTGCGGAGAT
bax	F-TCCACCAAGAAGCTGAGCGAG	R-GTCCAGCCCATGATGGTTCT
c-myc	F-GGTCTTCCCCTACCCTCTCA	R-CTCCAGCAGAAGGTGATCCA
GAPDH	F-GCTCTCTGCTCCTCCTGTTC	R-GTTGACTCCGACCTTCACCT

### NLRP3 activation and treatment with dexamethasone or IL-18 for lymphoma cells

Human lymphoma cell line Pfeiffer was cultured in RPMI 1640 (Gibco, USA) supplemented with 10% fetal bovine serum (FBS, Gibco, USA) and incubated in humidified air in 5% CO_2_ at 37°C. To activate NLRP3 inflammasome, Pfeiffer cells were treated with LPS (1μg / mL) for 6hand ATP (5mmol /L) for 1h, and then cultured in fresh medium.

Dexamethasone was added into Pfeiffer cells with or without NLRP3 activation at different concentrations (0, 50, 100, 150, 200, 250, 300 μmol/L) for 48 h to determine the drug sensitivity. IL-18 (10 ng/mL) and anti-IL-18 (1μg /mL) were also added into Pfeiffer cells and cultured for 48 h. Then, the cells and supernatant were collected for the following detection.

### Cell proliferation analysis

After being treated for 48 h, cell proliferation was assessed using CCK8 (Beyotime, China) method according to the manufacturer’s instructions. All experiments were conducted in triplicate.

### Cell cycle analysis

Pfeiffer cells were fixed in 75% alcohol overnight at 4°C, resuspended with PBS containing 0.2 mg/mL RNaseA, and incubated at 37°C for 30 min. Then, the cells were added with propidium iodide (PI) solution (Beyotime, China), and incubated in the dark for another 30 min at 4°C. DNA content was then analyzed using Gallios flow cytometry (Beckman Coulter, CA, USA).

### Cell apoptosis analysis

Apoptosis was analyzed using the Annexin V/PI apoptosis detection kit (Beyotime, China) according to the manufacturer’s instructions. Briefly, 3 × 10^5^ cells were resuspended in 400 μL of Annexin V binding buffer, and stained with 5 μl Annexin V in the dark for 15 min. Then, 10 μL PI was added and incubated for another 5 min. Stained cells were immediately measured by Gallios flow cytometry (Beckman Coulter, CA, USA).

### Enzyme-Linked Immunosorbent Assay (ELISA) for IL-18 and IL-1β

All plasma specimens or supernatants of cultured cells were obtained by centrifugation and stored at –80°C for determination of cytokines. The concentrations of IL-18 and IL-1 βwere determined with a quantitative sandwich enzyme immunoassay technique in accordance with the manufacturer’s recommendations (eBioscience, USA). The concentrations were calculated from a standard curve according to the manufacturer’s protocol.

### Western blot analysis

Cells were collected and lysed in the lysis buffer (Beyotime, China). After 15,000 g centrifugation for 15 min, the protein content of the supernatant was determined by an enhanced BCA protein assay Kit (Beyotime, China). The protein lysates were separated by 10% SDS-PAGE and subsequently electrotransferred onto a nitrocellulose filter membrane. The membrane was blocked with 5% nonfat milk for 2 h at room temperature. The blocked membrane was incubated with the indicated primary antibodies against ASC (A25291309, Adipogen, USA ) and NFκB65 (D14E12, Cell signal technology, USA) along with the reference antibody GAPDH (AB-P-R 001, Goodhere, China ), followed by incubation with a secondary HRP-conjugated antibody. The probed proteins were detected using enhanced chemiluminescent reagents (Millipore, USA). Protein bands were visualized using Western blotting detection system according to the manufacturer’s instructions. The results were quantified using the Image J software (National Institutes of Health, USA). For the quantification of specific bands, the same size square was drawn around each band to measure the intensity and then the value was adjusted by the intensity of the background near that band. The results were expressed as a relative ratio of the target protein to reference protein GAPDH.

### Statistical analysis

Continuous variables were compared with the one-way analysis of variance or nonparametric Kruskal-Wallis test, and categorical variables. Standard chi-square test was used to test genotype frequencies for Hardy-Weinberg equilibrium. Associations between genotype and disease risk were assessed by calculating odds ratios (OR) and corresponding 95% confidence intervals (CI). Group comparisons for the expression of mRNA and ELISA analyses were performed using Mann-Whitney *U* test. Kaplan-Meier curves were used to describe the survival. Cells experiments were performed three times and quantitative data are expressed as mean ± SD. Statistical analyses were performed using unpaired *t* test. Statistical analyses were conducted using Statistical Analysis System (SAS, USA) and GraphPad Prism 6.0 system. *P* value < 0.05 was considered statistically significant.
